# Effect of Glutamine Dipeptide Supplementation on Primary Outcomes for Elective Major Surgery: Systematic Review and Meta-Analysis

**DOI:** 10.3390/nu7010481

**Published:** 2015-01-09

**Authors:** Marta Sandini, Luca Nespoli, Massimo Oldani, Davide Paolo Bernasconi, Luca Gianotti

**Affiliations:** 1 Department of Surgery and Translational Medicine, Milano-Bicocca University, San Gerardo Hospital (4° piano A), via Pergolesi 33, 20900 Monza, Italy; E-Mails: sandinimarta84@gmail.com (M.S.); luca.nespoli@unimib.it (L.N.); oldanimassimo@gmail.com (M.O.); 2 Department of Health Sciences, Center of Biostatistics for Clinical Epidemiology, Milano-Bicocca University, 20900 Monza, Italy; E-Mail: davide_bernasconi@libero.it; 3 International Research Center in Hepato-Biliary-Pancreatic Diseases, 20900 Monza, Italy

**Keywords:** glutamine, dipeptide, surgery, outcome, infections, meta-analysis

## Abstract

To evaluate if glutamine (GLN) supplementation may affect primary outcomes in patients undergoing major elective abdominal operations, we performed a systematic literature review of randomized clinical trials (RCTs) published from 1983 to 2013 and comparing intravenous glutamine dipeptide supplementation to no supplementation in elective surgical abdominal procedures. A meta-analysis for each outcome (overall and infectious morbidity and length of stay) of interest was carried out. The effect size was estimated by the risk ratio (RR) or by the weighted mean difference (WMD). Nineteen RCTs were identified with a total of 1243 patients (640 receiving GLN and 603 controls). In general, the studies were underpowered and of medium or low quality. GLN supplementation did not affect overall morbidity (RR = 0.84, 95% CI 0.51 to 1.36; *p* = 0.473) and infectious morbidity (RR = 0.64; 95% CI = 0.38 to 1.07; *p* = 0.087). Patients treated with glutamine had a significant reduction in length of hospital stay (WMD = −2.67; 95% CI = −3.83 to −1.50; *p* < 0.0001). In conclusion, GLN supplementation appears to reduce hospital stay without affecting the rate of complications. The positive effect of GLN on time of hospitalization is difficult to interpret due to the lack of significant effects on surgery-related morbidity.

## 1. Introduction

Complications after major abdominal surgery remain a foremost concern based on their considerable impact on short- and long-term outcomes, quality of life, and the healthcare associated costs [1,2]. The pathogenesis of postoperative morbidity is multifactorial. Among the recognized factors are the effect of surgical trauma on protein turnover and synthesis, insulin resistance, immune response, gut permeability, organ function, and alterations of several others homeostatic and metabolic pathways [3,4]. The supplementation of key nutrients with pharmacological properties has been shown to partially restore these functions and improve patient outcome [5,6,7,8]. Glutamine is involved in a variety of biological processes, such as anabolic functions, acid-base regulation in the kidney, and ammonium metabolism [9]. Depletion in glutamine storage during stressful events [10,11] has been reported, and an exogenous supplementation is associated with improved protein synthesis, preservation of gut barrier, enhancement of wound healing, reduction of oxidative stress, negative nitrogen balance, improvement of glucose metabolism, and modulation of the immune system [12,13,14,15,16]. Whether this positive effect on surrogate endpoints translates into a protection of the occurrence of surgical-related complications is unclear. Some randomized clinical trials (RCTs) [17,18,19] reported a significant benefit of glutamine supplementation on postoperative morbidity while others did not confirm the results [20,21,22]. Such contrasting results had also been confirmed by previous meta-analyses [23,24,25,26]. These findings may be affected by several features such as study design, type of patients, baseline disease and nutritional status, concomitants treatments, and other factors that may influence differences in the results obtained in various studies. To help clinicians make decisions, it is necessary to summarize the controversial findings of various studies concerning the consequences of glutamine supplementation.

Thus, this study aims to review the findings of all available RCTs assessing the effect of additional parenteral glutamine on other treatments in a cohort of elective surgical patients candidate to major abdominal surgery and to analyze the data with a meta-analytic approach.

## 2. Materials and Methods

To conduct this research we followed the guidelines and the PRISMA statement for reporting systematic reviews and meta-analyses of studies evaluating healthcare interventions [27].

### 2.1. Literature Search

Two authors (MS, LN) independently performed a Medline, Embase, Pubmed, Scopus, Ovid, ISI Web of Science, and Cochrane Central Register of Controlled Trials and Cochrane Library database extended literature search of all studies published as original articles between March 1979 and December 2013. The following medical subject heading terms and words were used for the search, in all possible combinations: “glutamine”, “dipeptide”, “l-glutamine”, “nutritional support”, “artificial nutrition”, “enteral nutrition”, “parenteral nutrition”, “immunonutrition”, “pharmaconutrition” AND “abdominal”, “laparotomy”, “surgery”, “elective surgery”, “operations”, “procedure”.

The “related article” function was used to expand the search and the reference lists of articles selected for full-text review were searched for additional articles. In the event of overlap of authors, institutions or patients, the most recent or highest quality article was chosen.

### 2.2. Study Selection

The term “glutamine supplementation” was defined as any intravenous treatment that contains glutamine dipeptide alone or in combination with any form of artificial nutrition as reported in the articles reviewed.

Study inclusion criteria for eligibility were: adult population (age > 18 years), parallel-group RCTs reporting parenteral glutamine supplementation in patients who underwent major elective abdominal surgery and reporting at least one of the outcomes considered in the meta-analysis. We considered all studies irrespectively of whether intravenous glutamine was given in association with parenteral nutrition, enteral feeding or simple intravenous fluids or whether the control group received isonitrogenous/isocaloric regimens, whereas if glutamine was combined with other nutrients with potential immunometabolic activity (*i.e.*, arginine, nucleotides, omega-3 fatty acids), the studies were excluded. No full-text available articles, opinion pieces, editorials or papers written in languages other than English and German were included.

### 2.3. Data Extraction

An electronic database was created to collect all relevant trial data. The data were extracted independently by two investigators (MS, LN) and in case of disagreement an impartial rater (LG) cross-examined doubtful data and the decision was made after a consensus meeting.

Information extracted from the trials involved: first author, country of origin, year of publication, number of patients randomized, type of surgery, nutritional status and type of nutritional support, glutamine dosage and period of supplementation, regimen of the control groups, intention-to-treat reporting, double, single or no blindness, calculated study sample size, and the different outcome measures.

The primary purpose of this meta-analysis was to evaluate whether glutamine supplementation could affect surgery-related outcome measures. As primary relevant outcomes we assessed the rate of postoperative mortality, the rate of all postoperative complications and the rate of infectious morbidity. As secondary endpoint of the analysis we considered length of in-hospital stay after operation (LOS).

Study quality was assessed by two independent reviewers (LG, MO) according to a modified Jadad score [23] with a range from 0 to 7. No disagreement was reported.

### 2.4. Statistical Analysis

We performed a random-effects meta-analysis [28] for each outcome of interest. The effect size of mortality was estimated by the risk difference (RD) due to the presence of many studies with no deaths in both groups, for the other categorical outcomes (overall morbidity, infectious morbidity) the effect size was estimated by the risk ratio (RR), while for LOS the mean difference (MD) was used. In the calculation of the pooled risk ratio, a correction factor of 0.5 was added to all cell frequencies of studies where no patient had the outcome in either GLN or control groups [29]. Mean and standard deviation of length of stay was calculated according to method of Hozo *et al.* [30] for the studies where only median and range (or interquartile range) were reported. The weights assigned to each study were computed according to the inverse of the variance. Heterogeneity was quantified using I-squared and tau-squared indexes and testing the null hypothesis that all studies share a common effect size. Moreover, we investigated the presence of publication bias using funnel plots [31].

Finally, some stratified analyses were performed according to the following indicators: GLN dosage (>0.3 or ≤0.3 g/kg/day), duration of GLN supplement (>5 or or ≤5 days), intention to treat analysis (yes or not), blinding (yes or not), modified Jadad score (≥3 or <3), year of publication (>2002 or ≤2002), and type of surgery (lower gastrointestinal, upper gastrointestinal, or mixed procedures).

All the analyses were performed using “meta” package within R, version 3.0.2.

## 3. Results

**Figure 1:**
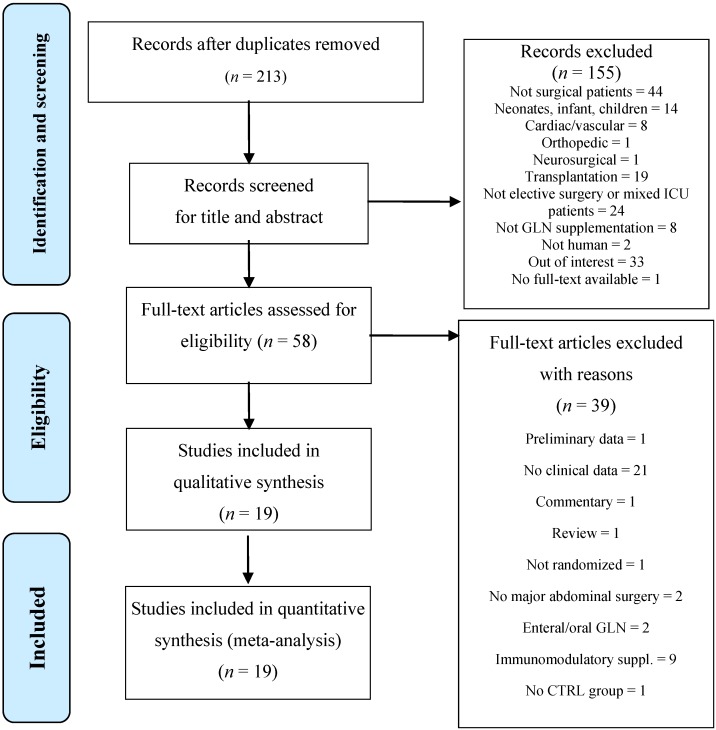
Flowchart of the literature search according the PRISMA statement.

The flow diagram of the literature search and article selection is depicted in Figure 1.

After removal of duplicates, we identified 213 potentially relevant references through the electronic searches. A total of 154 studies were excluded, as they were not on surgical patients (44), not referring to elective surgery candidates (24), on pediatric population (14), not regarding major abdominal surgery (10), irrelevant (43) or organ transplantation (19). We also excluded 25 trials because they did not provide information on clinical outcomes and seven in which glutamine was administrated in addition to other immunonutrients. It has been demonstrated that oral or enteral GLN administration has different effects with respect to the parenteral route, because they directly affect enterocyte activity before reaching the systemic circulation [32,33,34]. Given the different metabolic pathways of enteral glutamine, we decided to exclude the two trials left after the previous selection [35,36].

### 3.1. Study Characteristics

Nineteen RCTs were finally included in the meta-analysis; a total of 1243 patients, 640 (51.5%) receiving glutamine and 603 (48.5%) not receiving glutamine (control group). The mean number of patients per study was 65.42; most of the studies (68%) had 50 patients or less. Most RCTs were single center (16/19), 11 were double-blinded and only one was single blinded; sixteen trials (84%) reported ITT data; eight studies (42%) were conducted on cancer patients. In one trial the population was formed by malnourished patients [37], while in 13 trials the authors did not give information on the baseline nutritional status; nevertheless, in 79% (15/19) trials, patients were treated with total parenteral nutrition TPN.

The median GLN dosage was 0.3 g/kg/day.

Detailed information on all trials included in the meta-analysis is given in Table 1.

**Table 1:** Characteristics of the included trials.

Author	Year	Country	Population Analysed	Multi/Single Center	Blindness (Double/Single)	Type of Surgery	Cancer/Benign	Nutritional Status	GLN Dipeptide Dose (g/kg/day)	Artificial Nutrition (AN)	Type of AN	IC/IN Control	Onset	Study Power Calculation	ITT Morbidity	ITT LOS	Jadad Score
O’Riordain [38]	1994	UK	22	single	double	Lower GI	C/B	NA	0.18	Yes	TPN	YES	POD 1	NO	Yes	NA	3.5
Morlion [39]	1998	Germany	28	single	double	Lower GI	C/B	NA	0.30	Yes	TPN	YES	POD 1	NO	no	Yes	3.5
Jacobi [17]	1999	Germany	34	single	double	Upper GI	C/B	NA	0.40	Yes	TPN	YES	POD 1	NO	Yes	Yes	3.5
Jiang [40]	1999	China	60	multi	double	Mixed GI	C/B	NA	0.50	Yes	TPN	YES	POD 1	NO	Yes	Yes	5
Mertes [41]	2000	Germany	37	single	double	Mixed GI	NA	NA	0.50	Yes	TPN	YES	POD 1	NO	no	No	3
Karwowska [42]	2001	Poland	30	single	NO	Vascular	B	Well nourished	0.20	Yes	TPN	YES	POD 1	NO	Yes	Yes	1.5
Neri [43]	2001	Italy	33	single	double	Mixed GI	C	NA	0.30	Yes	TPN	YES	POD 1	NO	Yes	Yes	3
Spittler [37]	2001	Austria	30	single	NO	Mixed GI	NA	NA	0.50	NO	-	NA	POD 0	NO	Yes	Yes	2
Lin [20]	2002	China	48	single	double	Mixed GI	C	NA	0.42	Yes	TPN	YES	POD 1	NO	Yes	NA	4.5
Exner [21]	2003	Austria	45	single	NO	Mixed GI	NA	NA	0.50	Yes	TPN	YES	POD −1	NO	Yes	Yes	2.5
Klek [18]	2005	Poland	60	single	NO	Upper GI	C	Mixed	0.40	YES	TPN	YES	POD 1	NO	No	No	2
Yao [44]	2005	China	40	single	double	Mixed GI	C/B	NA	0.50	Yes	TPN	YES	POD −1	NO	Yes	Yes	5
Jo [22]	2006	Korea	60	single	double	Upper GI	C	Mixed	0.30	Yes	TPN	YES	POD −2	NO	Yes	Yes	4
Oguz [19]	2007	Turkey	109	single	NO	Lower GI	C	Mixed	1.00	Yes	EN	NA	POD −5	NO	No	No	2
Asprer [45]	2009	Philippines	34	multi	double	Mixed GI	C/B	Malnourished	0.30	Yes	mixed	YES	POD −5	NO	Yes	NA	5
Fan [46]	2009	China	40	single	NO	Mixed GI	C/B	NA	0.13	Yes	TPN	YES	POD −1	NO	Yes	Yes	2
Gianotti [47]	2009	Italy	428	multi	single	Mixed GI	C	Well nourished	0.40	NO	-	NA	POD −1	YES	Yes	Yes	5
Marton [48]	2010	Hungary	55	single	NO	Upper GI	C	NA	0.50	Yes	EN	YES	POD −3	YES	NA	NA	4
Lu [49]	2011	Taiwan	50	single	double	Mixed GI	C	NA	0.30	Yes	TPN	YES	POD 0	NO	Yes	NA	2.5

Legend: GLN: Glutamine; NA: not assessed; GI: gastrointestinal, IC/IN: isocaloric/isonitrogenous regimen, ITT: intention-to-treat, POD: postoperative or preoperative day.

### 3.2. Primary End-Points

**Figure 2:**
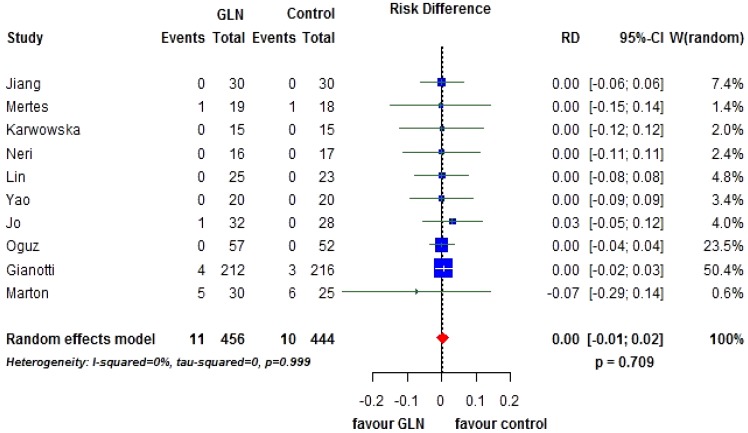
Forest plot for overall mortality comparing glutamine (GLN) *vs.* control. GLN: glutamine, RR: relative risk, CI: confidence interval.

**Figure 3:**
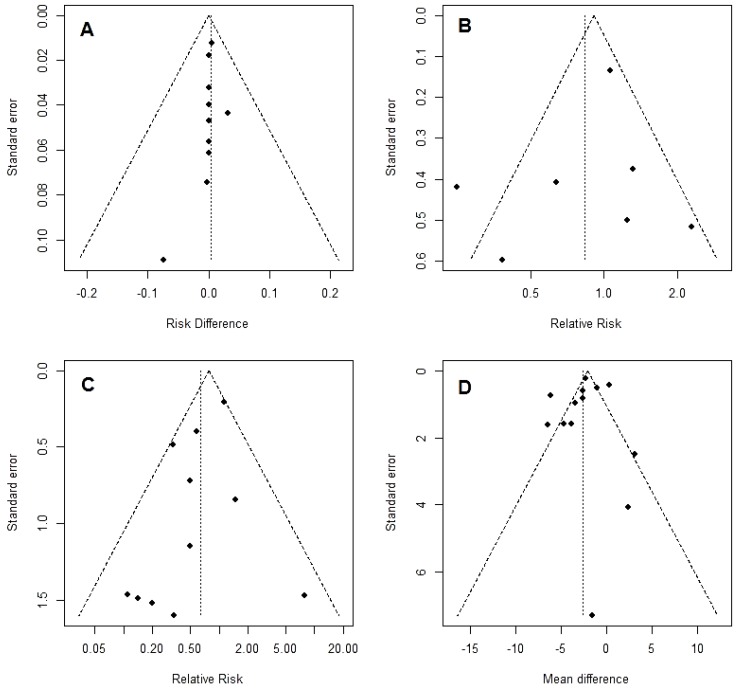
Funnel plot for mortality (**A**), overall morbidity (**B**), infectious morbidity (**C**) and length of in-hospital stay after operation (LOS) (**D**). For each study, the estimated effect measure is plotted on the x-axis and its standard error on the y-axis.

Ten trials [19,20,22,40,41,42,43,44,47,48], including 900 patients, provided data on postoperative mortality. However, only in four studies at least one event was observed. The rate was 2.41% in the patients receiving glutamine and 2.25% in controls. The overall risk difference was 0 (95% CI −0.01 to 0.02; *p* = 0.709). Heterogeneity between studies was negligible (I^2^ = 0%, *p* = 0.999) (Figure 2). No evidence of publication bias was detectable (Figure 3A).

**Figure 4:**
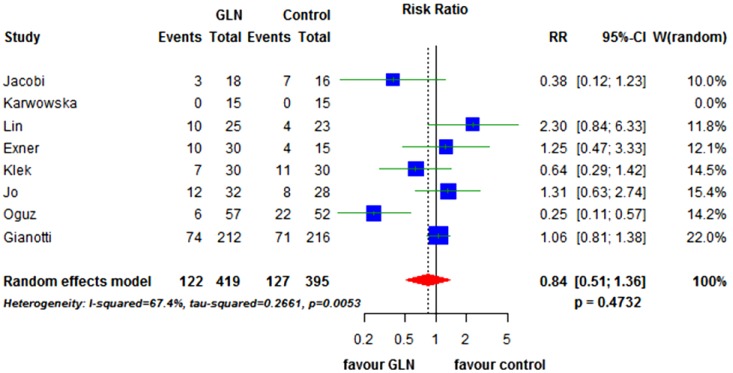
Forest plot for overall morbidity comparing GLN *vs.* control. GLN: glutamine, RR: relative risk, CI: confidence interval.

Eight RCTs [17,18,19,20,21,22,42,47] (814 patients) reported the overall rate of postoperative complications. The rate was 29.1% in patients receiving GLN *versus* 32.1% in controls. The effect of glutamine was not significant (RR = 0.84, 95% CI 0.51 to 1.36; *p* = 0.473). The tau-squared test for heterogeneity between studies was significant (*p* = 0.005) and I^2^ was 67.4% (Figure 4). Even though too few studies are included to draw a conclusion, the funnel plot suggests no strong evidence of publication bias (Figure 3B).

**Figure 5:**
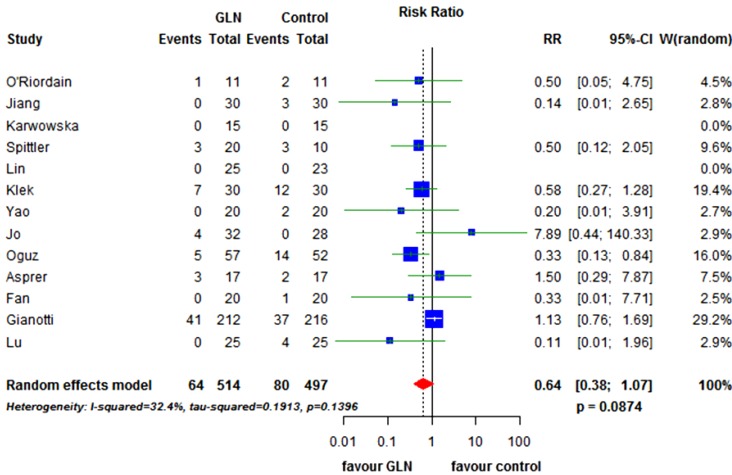
Forest plot for infectious morbidity comparing GLN *vs.* control. GLN: glutamine, RR: relative risk, CI: confidence interval.

Infectious morbidity was described in 13 trials [18,19,20,22,37,38,40,42,44,45,46,47,49], including 1011 patients. The rate was 12.6% in GLN *versus* 16.1% in controls. We observed a not significant effect of glutamine supplementation (RR = 0.64, 95% CI 0.38 to 1.07; *p* = 0.087). A moderate but not significant (*p* = 0.14) amount of heterogeneity between studies was present I^2^ = 32.4% (Figure 5). Observing the funnel plot, a slightly asymmetry toward the left can be hypothesized suggesting a possible publication bias in favor of studies with a positive GLN effect (Figure 3C).

### 3.3. Secondary End-Points

**Figure 6:**
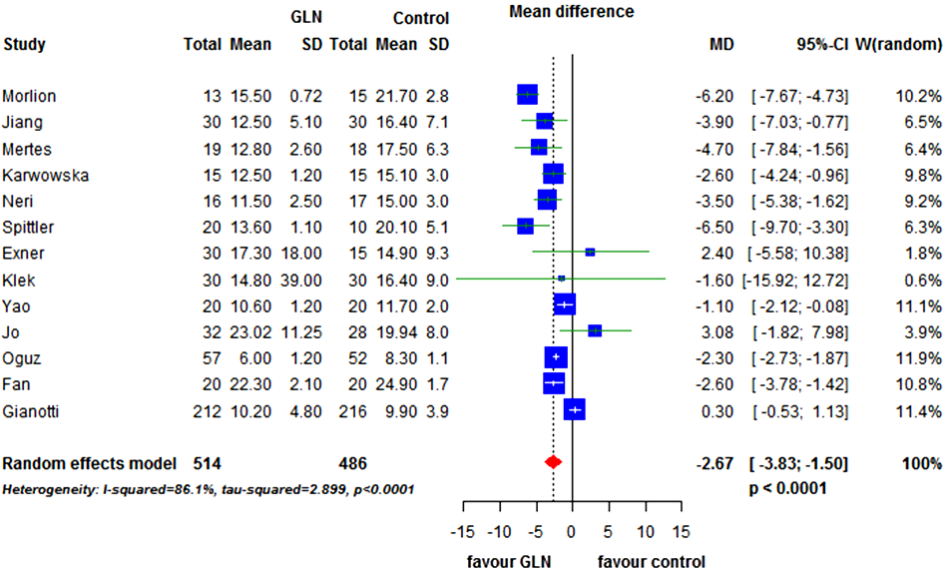
Forest plot for length of hospital stay comparing GLN *vs.* control. GLN: glutamine, MD: mean difference, CI: confidence interval.

Thirteen studies [18,19,21,22,39,40,41,42,43,44,46,47] (1000 patients) reported on LOS. There was a wide range of hospitalization time from a mean of 6 days to 25 days. The weighted mean difference was in favor of the patients receiving glutamine of −2.67 days (95% CI −3.83 to −1.50; *p* < 0.0001) (Figure 6).

There was significant heterogeneity between studies (I^2^ = 86.1%; *p* < 0.0001).

The funnel plot, shown in Figure 3D, did not clearly suggest the presence of publication bias.

### 3.4. Subgroup Analysis

We performed different subgroup analyses to evaluate possible influences of glutamine daily dosage (greater than 0.3 g/kg/day *versus* less or equal to 0.3 g/kg/day), period of supplementation (more than 5 days *versus* less or equal to 5 days), data reporting with or without intention-to-treat (ITT), blindness, quality of trials (modified Jadad score greater or equal to three points *versus* less than three points)), year of publication (before or during 2002 *versus* after 2002), and type of surgery (lower gastrointestinal, upper gastrointestinal, or mixed procedures).

A significant protective effect of GLN on overall morbidity (Table 2) was observed in only one study [19] with selective data reporting and enrolling patients with lower GI procedures.

The rate of infectious complications was significantly lower (RR = 0.46, 95% CI 0.25 to 0.84; *p* = 0.012) in patients treated with glutamine in trials [17,18] with selective data reporting (non ITT trials) and in studies [18,19,38,42,46,49], with a Jadad score lower than three (RR = 0.44, 95% CI 0.26 to 0.75; *p* = 0.0027) and in trials enrolling patients with lower GI procedures (RR = 0.35, 95% CI 0.14 to 0.83; *p* = 0.0179). This protective effect of GLN on postoperative infection rate was not confirmed in the analysis of trials with ITT reporting (RR = 0.81, 95% CI 0.45 to 1.46; *p* = 0.486), in studies with a Jadad score > three points (RR = 1.03, 95% CI 0.57 to 1.87; *p* = 0.916) and in trials with upper GI tract operations where the risk of infection was slightly increased (RR = 1.53) in patients with GLN supplementation (Table 3).

**Table 2:** Stratified analysis for overall morbidity comparing GLN *vs.* control.

Category	Study Characteristic (Number of Studies)	Overall RR (95% CI)	*p*-Value	I^2^%	*p*-Value for Heterogeneity between Strata
**Dosage of GLN**	>0.3 g per kg per day (6)	0.77 (0.43; 1.37)	0.3688	71.6	0.0035
	≤0.3 g per kg per day (1)	1.31 (0.63; 2.74)	0.4695	-	-
**Duration of GLN supplement**	>5 days (5)	0.87 (0.48; 1.57)	0.6383	74.5	0.0035
	≤5 days (2)	0.72 (0.23; 2.31)	0.5831	57	0.1271
**Intention to treat**	Yes (4)	1.11 (0.69; 1.78)	0.6713	45	0.1411
	No (1)	0.25 (0.11; 0.57)	0.0009	-	-
**Blinding**	Yes (3)	1.11 (0.45; 2.74)	0.8241	62.4	0.0699
	No (4)	0.70 (0.35; 1.38)	0.2974	75.7	0.0063
**Jadad score**	≥3 (4)	1.11 (0.69; 1.78)	0.6713	45	0.1411
	<3 (3)	0.57 (0.23; 1.39)	0.2165	69.3	0.0384
**Year of publication**	>2002 (5)	0.80 (0.46; 1.37)	0.4064	69.6	0.0105
	≤2002 (2)	0.96 (0.17; 5.59)	0.9640	80.7	0.0229
**Type of surgery**	Lower GI (1)	0.25 (0.11; 0.57)	0.0009	-	-
	Upper GI (3)	0.75 (0.38; 1.49)	0.4166	44.4	0.1655
	Mixed GI or vascular (3)	1.12 (0.85; 1.56)	0.3596	7.3	0.3401

For each category, the sum of the studies does not add up to the total number of studies considered due to missing information regarding overall morbidity. Studies with zero counts in both groups were excluded from the analysis. Legend: GLN: glutamine, RR: relative risk, CI: confidence interval, GI: Gastrointestinal.

**Table 3:** Stratified analysis for infectious morbidity comparing GLN *vs.* control.

Category	Study Characteristic (Number of Studies)	Overall RR (95% CI)	*p*-Value	I^2^%	*p*-Value for Heterogeneity between Strata
**Dosage of GLN**	>0.3 g per kg per day (6)	0.58 (0.32; 1.06)	0.0782	47.7	0.0887
	≤0.3 g per kg per day (5)	0.83 (0.24; 2.88)	0.77	23.6	0.2639
**Duration of GLN supplement**	>5 days (7)	0.61 (0.30; 1.23)	0.1677	53	0.0470
	≤5 days (4)	0.64 (0.26; 1.62)	0.3472	0	0.6211
**Intention to treat**	Yes (9)	0.81 (0.45; 1.46)	0.486	15.5	0.3043
	No (2)	0.46 (0.25; 0.84)	0.0119	0	0.3484
**Blinding**	Yes (6)	0.61 (0.18; 2.02)	0.4135	28.7	0.2196
	No (5)	0.64 (0.36; 1.14)	0.1329	47.3	0.1078
**Jadad score**	≥3 (6)	1.03 (0.57; 1.87)	0.9155	10.1	0.3509
	<3 (5)	0.44 (0.26; 0.75)	0.0027	0	0.7490
**Year of publication**	>2002 (8)	0.69 (0.37; 1.28)	0.2391	44.8	0.0802
	≤2002 (3)	0.42 (0.14; 1.26)	0.1218	0	0.7205
**Type of surgery**	Lower GI (2)	0.35 (0.14; 0.83)	0.0179	0	0.7310
	Upper GI (2)	1.53 (0.11; 21.38)	0.7504	70.1	0.0674
	Mixed GI or vascular (7)	0.75 (0.40; 1.41)	0.3772	17.4	0.2969

For each category, the sum of the studies does not add up to the total number of studies considered due to missing information regarding infectious morbidity. Studies with zero counts in both groups were excluded from the analysis. Legend: GLN: glutamine, RR: relative risk, CI: confidence interval, GI: gastrointestinal.

**Figure 7:**
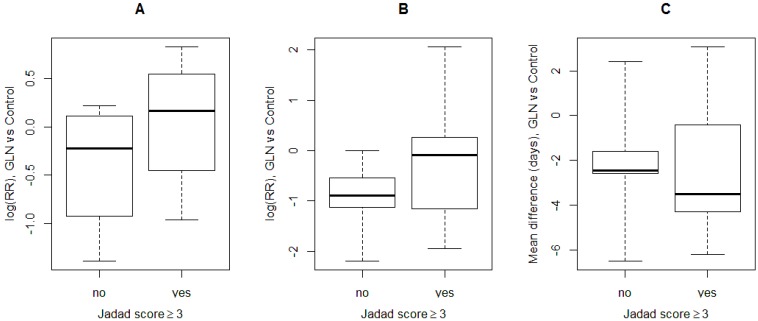
Box-plots comparing the distribution of the GLN effect between high quality (Jadad score ≥ 3) and low quality studies (Jadad score < 3) for overall morbidity (**A**), infectious morbidity (**B**) and length of stay (**C**). GLN: glutamine, RR: relative risk, log: natural logarithm.

The difference between high- and low-quality studies in the assessment of the effect of GLN on postoperative complications and LOS was further explored by a boxplot graph (Figure 7). The effect of GLN on overall morbidity and infectious morbidity was overestimated in low quality studies, where the distribution of the log RR lay almost totally below zero, compared to high quality studies, where the distribution was centered around zero. This was not true for LOS, where the distribution of the GLN effect in both study groups was located in the same range.

The significant reduction in LOS in groups receiving glutamine supplementation was confirmed in all subgroup analyses except for upper GI surgery (Table 4).

**Table 4:** Stratified analysis for length of hospital stay comparing GLN *vs.* control.

Category	Study Characteristic (Number of Studies)	Overall MD (95% CI)	*p*-Value	I^2^%	*p*-Value for Heterogeneity between Strata
**Dosage of GLN**	>0.3 g per kg per day (8)	−2.26 (−3.71; −0.81)	0.0023	84.8	<0.0001
	≤0.3 g per kg per day (5)	−3.06 (−4.99; −1.13)	0.0019	82.8	0.0001
**Duration of GLN supplement**	>5 days (8)	−1.95 (−3.19; −0.71)	0.002	82.8	<0.0001
	≤5 days (5)	−3.85 (−6.96; −0.73)	0.0155	89.7	<0.0001
**Intention to treat**	Yes (10)	−2.53 (−4.16; 0.89)	0.0024	89	<0.0001
	No (3)	−2.54 (−3.59; −1.49)	<0.0001	9.8	0.3299
**Blinding**	Yes (6)	−3.06 (−5.40; −0.72)	0.0103	87.2	<0.0001
	No (7)	−2.16 (−3.60; −0.71)	0.0034	85.5	<0.0001
**Jadad score**	≥3 (7)	−2.48 (−4.66; −0.31)	0.0252	91.7	<0.0001
	<3 (6)	−2.66 (−3.53; −1.78)	<0.0001	38.2	0.1512
**Year of publication**	>2002 (7)	−1.08 (−2.38; 0.23)	0.1067	84.2	<0.0001
	≤2002 (6)	−4.47 (−5.93; −3.01)	<0.0001	61.7	0.0228
**Type of surgery**	Lower GI (2)	−4.18 (−8.00; −0.36)	0.0318	96	<0.0001
	Upper GI (2)	2.59 (−2.04; 7.22)	0.2732	0	0.5445
	Mixed GI or vascular (9)	−2.55 (−3.93; −1.18)	0.0003	82.1	<0.0001

For each category, the sum of the studies does not add up to the total number of studies considered due to missing information about length of stay. Legend: GLN: glutamine, MD: mean difference, CI: confidence interval, GI: gastrointestinal.

## 4. Discussion

The results gathered from the present meta-analysis suggest that parenteral glutamine supplementation given to patients undergoing major elective abdominal surgery do not significantly affect primary clinical endpoints such as mortality, overall morbidity, and the occurrence of infectious complications. We observed a reduction in postoperative infection rate in the glutamine supplemented group in the subgroup analyses only in the non-ITT analysis (two RCTs), in studies with low quality (five RCTs) and in trials with patients operated on the lower GI tract (two RCTs). In contrast, patients undergoing upper GI procedures had a trend toward an increased risk of infections if treated with glutamine. These results need to be evaluated with caution for the relative small number of trials with these specific inclusion criteria, most of the studies having been performed on a mixed or undefined types of operations.

The lack of agreement of our results with previous meta-analyses [23,24,25,26] may be based on several reasons. The first meta-analysis by Novak *et al.* [24] was published in 2002 and the second by Zheng in 2006 [25]. Since than another seven RCTs [19,22,45,46,47,48,49] have become available in the literature and, therefore, the different conclusions may be justified by the number of trials analyzed. Another two more recent meta-analyses published in 2010 [26] and 2013 [23] suggested that glutamine supplementation in this specific cohort of patients was associated with a significant better outcome as measured by postoperative infection rate and LOS. Wang [26] included 447 patients enrolled in 10 trials reporting on infectious morbidity, and the study search was interrupted in October 2008. Since then, another five RCTs have been published [46,47,48,49] and the overall number of patients considerable for analysis has more than doubled. In the latest meta-analysis investigating the role of parenteral glutamine, published by Bollhalder *et al.* [23], a significant protective effect of treatment on infectious morbidity was observed for RCTs on surgical patients (risk ratio of 0.61, 95% CI 0.46 to 0.82). These authors defined surgical patients as all subjects candidate to any operation including emergency cases while we selected just elective procedures. In particular, in comparison to Bollhalder *et al.* [23], we excluded five trials [50,51,52,53,54], the first three for emergency cases, the fourth because of lack of randomization and the last one because of inclusion of non-abdominal surgery. It is reasonable to expect that patients operated for emergent diseases have a non comparable risk of developing infections or overall morbidity with respect to elective procedures.

The study by Karwowska *et al.* was included in the present analysis because the patients enrolled underwent elective laparotomic aneurism repair. A potential mechanism of postoperative infections in these subjects is the increased bacterial translocation due to a leaky gut [55,56,57] and glutamine supplementation has been shown to be effective in this scenario [58].

Yet, we evaluated additional three trials [19,47,48] in which glutamine supplementation was not associated with TPN. The rational to do so is that the administration of immunonutrients has been shown to have a protective effect regarding surgical-related complications regardless of the patient baseline nutritional status [59,60,61] for the well-established effect of key nutrients dissociated from nutritional support [62]. Most of the patients enrolled in the RCTs considered in the present meta-analysis were treated with TPN even if they were well-nourished or if the baseline nutritional status was not stated, possibly underlining an overuse of parenteral nutrition. Parenteral overfeeding as an additional risk factor for postoperative infectious complications has been emphasized in recent guidelines [63]. It may be speculated that the lower rate of infections in the glutamine groups, when both treated and control groups received TPN, is to be interpreted not as a primary protective effect of glutamine but rather as a masking outcome of the detrimental effect of TPN overuse. Another potential confounding factor in most of the trials was the necessity of altering a balanced amino acid composition in the treated group to obtain an isonitrogenous regimen with respect to the controls. This could have resulted in a deficiency of important amino acids in the patients receiving glutamine supplementation.

Our analysis confirmed previous findings [23,24,25,26] on the protective effect of glutamine supplementation on length of hospital stay in surgical patients. This observation seems difficult to interpret for several reasons. Firstly because a shorter LOS may be considered a reliable parameter of recovery and outcome only when consistent with improved morbidity. Secondly because LOS is trustworthy only when *a priori* definition of the discharge criteria are clearly stated, and none of the trials examined had this feature. Otherwise this parameter may be highly dependent on subjective assessment and influenced by non-clinical parameters, such as social and economical conditions, department organization, practice style, and type of primary care provider [64]. Furthermore, paradoxically we observed that in subgroup analyses preformed in patients with lower glutamine doses and shorter periods of administration, an equal or higher rate of complication with respect to groups receiving higher dose and longer time was found. Yet, LOS of the first two types of treatment were shorter than in the others groups. Moreover, this effect was confirmed in a subgroup analysis of high quality studies.

LOS is a relevant endpoint to weight because reduction of hospitalization time may have an impact on healthcare resources. Nevertheless, Taheri *et al.* [65] showed that the expenses directly attributable to the last days of a hospital stay are an economically insignificant component of total costs of the clinical process. Maybe physicians and administrators should deemphasize LOS and focus instead on process changes that use capacity and alter care delivery during the other stages of admission and on avoidance of complications when resource consumption is most intense. In this perspective, it is essential in future trials to focus on robust primary endpoints rather than on LOS to evaluate the effect of treatments on recovery and outcome unless precise criteria of hospital discharge are defined [66].

A shortcoming of our meta-analysis as other ones is the inclusion of trials with small sample sizes. We observed that all but two trials did not give information on the study power, and in most of the studies the number of patients enrolled per trial was less than 50. It has been implied that results from small trials should be cautiously weighed up because they are supposed to overestimate the expected effect of treatment [67]. Moreover, evidence suggests that meta-analysis run on underpowered studies may be potentially unreliable in various medical and surgical settings [68,69,70,71].

Concerning the overall effects of the meta-analysis, it is worth noting that there is more power to test a difference in LOS than in detecting an effect on the other endpoints, both because LOS is a continuous variable and because more studies report data on LOS. However, after performing some power calculations for overall morbidity and infectious morbidity computed using the random effects model [72], we observe that even assuming a large heterogeneity (between-study variance as large as within-study variance) the power of detecting even a small effect size (0.30) is high (>80%).

With respect to the analysis of the overall rate of morbidity, it should be underlined that most of the studies did not report details on the type and severity of complications creating a potential bias in our results. Lastly, to lower the risk of bias, we grouped the only single blinded RCT with the non-blinded ones, because single blinding has a lower methodological value than double blinding.

## 5. Conclusions

Our findings do not support routine glutamine supplementation in patients undergoing elective major abdominal surgery. We cannot exclude that in selected cohorts of high risk patients, such as those severely malnourished, glutamine may be useful in improving primary outcomes.

## References

[1] Vonlanthen R., Slankamenac K., Breitenstein S., Puhan M.A., Muller M.K., Hahnloser D., Hauri D., Graf R., Clavien P.-A. (2011). The impact of complications on costs of major surgical procedures: A cost analysis of 1200 patients. Ann. Surg..

[2] Braga M., Gianotti L., Vignali A., Schmid A., Nespoli L., Di Carlo V. (2005). Hospital resources consumed for surgical morbidity: Effects of preoperative arginine and omega-3 fatty acid supplementation on costs. Nutrition.

[3] Marik P.E., Flemmer M. (2012). The immune response to surgery and trauma: Implications for treatment. J. Trauma Acute Care Surg..

[4] Hill G.L., Douglas R.G., Schroeder D. (1993). Metabolic basis for the management of patients undergoing major surgery. World J. Surg..

[5] Senkal M., Kemen M., Homann H.H., Eickhoff U., Baier J., Zumtobel V. (1995). Modulation of postoperative immune response by enteral nutrition with a diet enriched with arginine, RNA, and omega-3 fatty acids in patients with upper gastrointestinal cancer. Eur. J. Surg..

[6] Gianotti L., Braga M., Fortis C. (1999). A prospective randomized clinical trial on perioperative feeding with arginine, omega 3 fatty acids, and RNA enriched enteral diets: Effect on host response and nutritional status. JPEN J. Parenter. Enteral Nutr..

[7] Braga M., Gianotti L., Radaelli G., Vignali A., Mari G., Gentilini O., Di Carlo V. (1999). Perioperative immunonutrition in patients undergoing cancer surgery: Results of a randomized double-blind phase III trial. Arch. Surg..

[8] Marimuthu K., Varadhan K.K., Ljungqvist O., Lobo D.N. (2012). A meta-analysis of the effect of combinations of immune modulating nutrients on outcome in patients undergoing major open gastrointestinal surgery. Ann. Surg..

[9] Soeters P.B., Grecu I. (2012). Have we enough glutamine and how does it work? A clinician’s view. Ann. Nutr. Metab..

[10] Petersson B., Vinnars E., Waller S.O., Wernerman J. (1992). Long-term changes in muscle free amino acid levels after elective abdominal surgery. Br. J. Surg..

[11] Van Acker B.A., Hulsewé K.W., Wagenmakers A.J., Soeters P.B., von Meyenfeldt M.F. (2000). Glutamine appearance rate in plasma is not increased after gastrointestinal surgery in humans. J. Nutr..

[12] Kim H. (2011). Glutamine as an immunonutrient. Yonsei Med. J..

[13] Souba W.W., Klimberg V.S., Hautamaki R.D., Mendenhall W.H., Bova F.C., Howard R.J., Bland K.I., Copeland E.M. (1990). Oral glutamine reduces bacterial translocation following abdominal radiation. J. Surg. Res..

[14] Souba W.W., Klimberg V.S., Plumley D.A., Salloum R.M., Flynn T.C., Bland K.I., Copeland E.M. (1990). The role of glutamine in maintaining a healthy gut and supporting the metabolic response to injury and infection. J. Surg. Res..

[15] Braga M., Wischmeyer P.E., Dover J., Heyland D.K. (2013). Clinical evidence for pharmaconutrition in major elective surgery. JPEN J. Parenter. Enteral Nutr..

[16] Stehle P., Zander J., Mertes N., Albers S., Puchstein C., Lawin P., Fürst P. (1989). Effect of parenteral glutamine peptide supplements on muscle glutamine loss and nitrogen balance after major surgery. Lancet.

[17] Jacobi C.A., Ordemann J., Zuckermann H., Döcke W., Volk H.D., Müller J.M. (1999). The influence of alanyl-glutamine on immunologic functions and morbidity in postoperative total parenteral nutrition. Preliminary results of a prospective randomized trial. Zentralbl. Chir..

[18] Kłek S., Kulig J., Szczepanik A.M., Jedrys J., Kołodziejczyk P. (2005). The clinical value of parenteral immunonutrition in surgical patients. Acta Chir. Belg..

[19] Oguz M., Kerem M., Bedirli A., Mentes B.B., Sakrak O., Salman B., Bostanci H. (2007). l-alanin-l-glutamine supplementation improves the outcome after colorectal surgery for cancer. Colorectal Dis..

[20] Lin M.T., Kung S.P., Yeh S.L., Lin C., Lin T.H., Chen K.H., Liaw K.Y., Lee P.H., Chang K.J., Chen W.J. (2002). The effect of glutamine-supplemented total parenteral nutrition on nitrogen economy depends on severity of diseases in surgical patients. Clin. Nutr..

[21] Exner R., Tamandl D., Goetzinger P., Mittlboeck M., Fuegger R., Sautner T., Spittler A., Roth E. (2003). Perioperative GLY-GLN infusion diminishes the surgery-induced period of immunosuppression: Accelerated restoration of the lipopolysaccharide-stimulated tumor necrosis factor-alpha response. Ann. Surg..

[22] Jo S., Choi S.H., Heo J.S., Kim E.M., Min M.S., Choi D.W., Seo J.M., Chung J.C., Kim Y.I. (2006). Missing effect of glutamine supplementation on the surgical outcome after pancreaticoduodenectomy for periampullary tumors: A prospective, randomized, double-blind, controlled clinical trial. World J. Surg..

[23] Bollhalder L., Pfeil A.M., Tomonaga Y., Schwenkglenks M. (2013). A systematic literature review and meta-analysis of randomized clinical trials of parenteral glutamine supplementation. Clin. Nutr..

[24] Novak F., Heyland D.K., Avenell A., Drover J.W., Su X. (2002). Glutamine supplementation in serious illness: A systematic review of the evidence. Crit. Care Med..

[25] Zheng Y., Li F., Qi B., Luo B., Sun H., Liu S., Wu X. (2007). Application of perioperative immunonutrition for gastrointestinal surgery: A meta-analysis of randomized controlled trials. Asia Pac. J. Clin. Nutr..

[26] Wang Y., Jiang Z.M., Nolan M.T., Jiang H., Han H.R., Yu K., Li H.L., Jie B., Liang X.K. (2010). The impact of glutamine dipeptide-supplemented parenteral nutrition on outcomes of surgical patients: A meta-analysis of randomized clinical trials. JPEN J. Parenter. Enteral Nutr..

[27] Moher D., Liberati A., Tetzlaff J., Altman D.G., PRISMA Group (2009). Preferred reporting items for systematic reviews and meta-analyses: The PRISMA statement. PLoS Med..

[28] DerSimonian R., Laird N. (1986). Meta-analysis in clinical trials. Control Clin. Trials.

[29] Sweeting M.J., Sutton A.J., Lambert P.C. (2004). What to add to nothing? Use and avoidance of continuity corrections in meta-analysis of sparse data. Stat. Med..

[30] Hozo S., Djulbegovic B., Hozo I. (2005). Estimating the mean and variance from the median, range, and the size of a sample. BMC Med. Res. Methodol..

[31] Sterne J.A.C., Egger M. (2001). Funnel plots for detecting bias in meta-analysis: Guidelines on choice of axis. J. Clin. Epidemiol..

[32] Han T., Li X.L., Cai D.L., Zhong Y., Geng S.S. (2013). Effects of glutamine-supplemented enteral or parenteral nutrition on apoptosis of intestinal mucosal cells in rats with severe acute pancreatitis. Eur. Rev. Med. Pharmacol. Sci..

[33] Mc Anena O., Moore F.M., Moore E., Jones T., Parsons P. (1991). Selective uptake of glutamine in the gastrointestinal tract: Confirmation in a human study. Br. J. Surg..

[34] Peng X., Yan H., You Z., Wang P., Wang S. (2004). Effects of enteral supplementation with glutamine granules on intestinal mucosal barrier function in severe burned patients. Burns.

[35] Liu H., Ling W., Shen Z.Y., Jin X., Cao H. (2012). Clinical application of immune-enhanced enteral nutrition in patients with advanced gastric cancer after total gastrectomy. J. Dig. Dis..

[36] Quan Z.F., Yang C., Li N., Li J.S. (2004). Effect of glutamine on change in early postoperative intestinal permeability and its relation to systemic inflammatory response. World J. Gastroenterol..

[37] Spittler A., Sautner T., Gornikiewicz A., Manhart N., Oehler R., Bergmann M., Függer R., Roth E. (2001). Postoperative glycyl-glutamine infusion reduces immunosuppression: Partial prevention of the surgery induced decrease in HLA-DR expression on monocytes. Clin. Nutr..

[38] O’Riordain M.G., Fearon K.C., Ross J.A., Rogers P., Falconer J.S., Bartolo D.C., Garden O.J., Carter D.C. (1994). Glutamine-supplemented total parenteral nutrition enhances T-lymphocyte response in surgical patients undergoing colorectal resection. Ann. Surg..

[39] Morlion B.J., Stehle P., Wachtler P., Siedhoff H.P., Köller M., König W., Fürst P., Puchstein C. (1998). Total parenteral nutrition with glutamine dipeptide after major abdominal surgery: A randomized, double-blind, controlled study. Ann. Surg..

[40] Jiang Z.M., Cao J.D., Zhu X.G., Zhao W.X., Yu J.C., Ma E.L., Wang X.R., Zhu M.W., Shu H., Liu Y.W. (1999). The impact of alanyl-glutamine on clinical safety, nitrogen balance, intestinal permeability, and clinical outcome in postoperative patients: A randomized, double-blind, controlled study of 120 patients. JPEN J. Parenter. Enteral. Nutr..

[41] Mertes N., Schulzki C., Goeters C., Winde G., Benzing S., Kuhn K.S., Van Aken H., Stehle P., Fürst P. (2000). Cost containment through l-alanyl-l-glutamine supplemented total parenteral nutrition after major abdominal surgery: A prospective randomized double-blind controlled study. Clin. Nutr..

[42] Karwowska K.A., Dworacki G., Trybus M., Zeromski J., Szulc R. (2001). Influence of glutamine-enriched parenteral nutrition on nitrogen balance and immunologic status in patients undergoing elective aortic aneurysm repair. Nutrition.

[43] Neri A., Mariani F., Piccolomini A., Testa M., Vuolo G., Di Cosmo L. (2001). Glutamine-supplemented total parenteral nutrition in major abdominal surgery. Nutrition.

[44] Yao G.X., Xue X.B., Jiang Z.M., Yang N.F., Wilmore D.W. (2005). Effects of perioperative parenteral glutamine-dipeptide supplementation on plasma endotoxin level, plasma endotoxin inactivation capacity and clinical outcome. Clin. Nutr..

[45] Asprer J.M., Llido L.O., Sinamban R., Schlotzer E., Kulkarni H. (2009). Effect on immune indices of preoperative intravenous glutamine dipeptide supplementation in malnourished abdominal surgery patients in the preoperative and postoperative periods. Nutrition.

[46] Fan Y.P., Yu J.C., Kang W.M., Zhang Q. (2009). Effects of glutamine supplementation on patients undergoing abdominal surgery. Chin. Med. Sci. J..

[47] Gianotti L., Braga M., Biffi R., Bozzetti F., Mariani L. (2009). Perioperative intravenous glutamine supplemetation in major abdominal surgery for cancer: A randomized multicenter trial. Ann. Surg..

[48] Marton S., Ghosh S., Papp A., Bogar L., Koszegi T., Juhasz V., Cseke L., Horvath P.O. (2010). Effect of glutamine in patients with esophagus resection. Dis. Esophagus.

[49] Lu C.Y., Shih Y.L., Sun L.C., Chuang J.F., Ma C.J., Chen F.M., Wu D.C., Hsieh J.S., Wang J.Y. (2011). The inflammatory modulation effect of glutamine-enriched total parenteral nutrition in postoperative gastrointestinal cancer patients. Am. Surg..

[50] Goeters C., Wenn A., Mertes N., Wempe C., van Aken H., Stehle P., Bone H.G. (2002). Parenteral l-alanyl-l-glutamine improves 6-month outcome in critically ill patients. Crit. Care Med..

[51] Fuentes-Orozco C., Anaya-Prado R., Gonzalez-Ojeda A., Arenas-Marquez H., Cabrera-Pivaral C., Cervantes-Guevara G., Barrera-Zepeda L.M. (2004). l-alanyl-l-glutamine-supplemented parenteral nutrition improves infectious morbidity in secondary peritonitis. Clin. Nutr..

[52] Estivariz C.F., Griffith D.P., Luo M., Szeszycki E.E., Bazargan N., Dave N., Daignault N.M., Bergman G.F., McNally T., Battey C.H. (2008). Efficacy of parenteral nutrition supplemented with glutamine dipeptide to decrease hospital infections in critically ill surgical patients. JPEN J. Parenter. Enteral Nutr..

[53] Yeh C.N., Lee H.L., Liu Y.Y., Chiang K.C., Hwang T.L., Jan Y.Y., Chen M.F. (2008). The role of parenteral glutamine supplement for surgical patient perioperatively: Result of a single center, prospective and controlled study. Langenbecks Arch. Surg..

[54] Engel J.M., Pitz S., Muhling J., Menges T., Martens F., Kwapisz M., Hempelmann G. (2009). Role of glutamine administration on T-cell derived inflammatory response after cardiopulmonary bypass. Clin. Nutr..

[55] Wilmore D.W., Smith R.J., O’Dwyer S.T., Jacobs D.O., Ziegler T.R., Wang X.D. (1988). The gut: A central organ after surgical stress. Surgery.

[56] Baue A.E. (1991). Nutrition and metabolism in sepsis and multisystem organ failure. Surg. Clin. N. Am..

[57] Alexander J.W. (1993). Immunonutrition: An emerging strategy in the ICU. J. Crit. Care Nutr..

[58] Gianotti L., Alexander J.W., Gennari R., Pyles T., Babcock G.F. (1995). Oral glutamine decreases bacterial translocation and improves survival in experimental gut-origin sepsis. JPEN J. Parenter. Enteral Nutr..

[59] Gianotti L., Braga M., Nespoli L., Radaelli G., Beneduce A., Di Carlo V. (2002). A randomized controlled trial of preoperative oral supplementation with a specialized diet in patients with gastrointestinal cancer. Gastroenterology.

[60] Braga M., Gianotti L., Nespoli L., Radaelli G., Di Carlo V. (2002). Nutritional approach in malnourished surgical patients: A prospective randomized study. Arch. Surg..

[61] Cerantola Y., Hübner M., Grass F., Demartines N., Schäfer M. (2011). Immunonutrition in gastrointestinal surgery. Br. J. Surg..

[62] Bozzetti F., Gianotti L., Braga M., Di Carlo V., Mariani L. (2007). Postoperative complications in gastrointestinal cancer patients: The joint role of the nutritional status and the nutritional support. Clin. Nutr..

[63] Braga M., Ljungqvist O., Soeters P., Fearon K., Weimann A., Bozzetti F. (2009). ESPEN Guidelines on Parenteral Nutrition: Surgery. Clin. Nutr..

[64] Krell R.W., Girotti M.E., Dimick J.B. (2014). Extended length of stay after surgery complications: Inefficient practice, or sick patients?. JAMA Surg..

[65] Taheri P.A., Butz D.A., Greenfield L.J. (2000). Length of stay has minimal impact on the cost of hospital admission. J. Am. Coll. Surg..

[66] Maessen J.M., Dejong C.H., Kessels A.G., von Meyenfeldt M.F., Enhanced recovery after surgery (ERAS) group (2008). Length of stay: An inappropriate readout of the success of enhanced recovery programs. World J. Surg..

[67] Royall R.M. (1986). The effect of sample size on the meaning of significance tests. Am. Stat..

[68] Kjaergard L.L., Villumsen J., Gluud C. (2001). Reported methodologic quality and discrepancies between large and small randomized trials in meta-analyses. Ann. Intern. Med..

[69] Reichenbach S., Sterchi R., Scherer M., Trelle S., Bürgi E., Bürgi U., Dieppe P.A., Jüni P. (2007). Meta-analysis: Chondroitin for osteoarthritis of the knee or hip. Ann. Intern. Med..

[70] Rerkasem K., Rothwell P.M. (2010). Meta-analysis of small randomized controlled trials in surgery may be unreliable. Br. J. Surg..

[71] Nüesch E., Trelle S., Reichenbach S., Rutjes A.W., Tschannen B., Altman D.G., Egger M., Jüni P. (2010). Small study effects in meta-analyses of osteoarthritis trials: Meta-epidemiological study. BMJ.

[72] Hedges L.V., Pigott T.D. (2001). The power of statistical tests in meta-analysis. Psychol. Methods.

